# MAD1-dependent recruitment of CDK1-CCNB1 to kinetochores promotes spindle checkpoint signaling

**DOI:** 10.1083/jcb.201808015

**Published:** 2019-01-23

**Authors:** Tatiana Alfonso-Pérez, Daniel Hayward, James Holder, Ulrike Gruneberg, Francis A. Barr

**Affiliations:** 1 Department of Biochemistry, University of Oxford, Oxford, UK; 2 Sir William Dunn School of Pathology, University of Oxford, Oxford, UK

## Abstract

Cyclin B–dependent kinase (CDK1-CCNB1) promotes entry into mitosis. Additionally, it inhibits mitotic exit by activating the spindle checkpoint. This latter role is mediated through phosphorylation of the checkpoint kinase MPS1 and other spindle checkpoint proteins. We find that CDK1-CCNB1 localizes to unattached kinetochores and like MPS1 is lost from these structures upon microtubule attachment. This suggests that CDK1-CCNB1 is an integral component and not only an upstream regulator of the spindle checkpoint pathway. Complementary proteomic and cell biological analysis demonstrate that the spindle checkpoint protein MAD1 is one of the major components of CCNB1 complexes, and that CCNB1 is recruited to unattached kinetochores in an MPS1-dependent fashion through interaction with the first 100 amino acids of MAD1. This MPS1 and MAD1-dependent pool of CDK1-CCNB1 creates a positive feedback loop necessary for timely recruitment of MPS1 to kinetochores during mitotic entry and for sustained spindle checkpoint arrest. CDK1-CCNB1 is therefore an integral component of the spindle checkpoint, ensuring the fidelity of mitosis.

## Abstract (precis)

CDK1-CCNB1 plays a well-established role in promoting mitosis. Alfonso-Pérez et al. now show that CDK1-CCNB1 forms a complex with the spindle checkpoint protein MAD1 at unattached kinetochores that directly promotes spindle checkpoint signaling.

## Introduction

In eukaryotic cells, accurate chromosome segregation requires the spindle assembly checkpoint, a surveillance system monitoring kinetochore attachment to microtubules of the mitotic spindle (Lara-Gonzalez et al., 2012; Musacchio, 2015). The spindle checkpoint kinase MPS1 binds to unattached kinetochores and phosphorylates kinetochore proteins, thus directing the accumulation of spindle checkpoint proteins of the MAD and BUB families (Musacchio, 2015; Ciliberto and Hauf, 2017). A subset of the MAD and BUB proteins then assemble into the mitotic checkpoint complex (MCC; Musacchio, 2015). The mitotic checkpoint complex then diffuses away from the kinetochore to inhibit the ubiquitin E3 ligase anaphase promoting complex/cyclosome (APC/C), thus preventing mitotic exit (Sivakumar and Gorbsky, 2015). The two crucial targets ubiquitylated by the APC/C to promote mitotic exit are securin, the inhibitor of separase, and most important for this work, cyclin B, the activating subunit of a cyclin-dependent mitotic kinase (CDK1). Destruction of cyclin B is delayed until metaphase by the spindle checkpoint (Li et al., 1997; Fang et al., 1998). In contrast, the related cyclin A is destroyed in prophase and prometaphase in a checkpoint-independent manner (Geley et al., 2001; Di Fiore and Pines, 2010). This indicates that distinct properties of cyclin A and B are required to initiate and then sustain mitosis (Gong and Ferrell, 2010). Most obviously, CDK2–cyclin A and CDK1­­–cyclin B show different localizations in cells (Minshull et al., 1990; Pines and Hunter, 1991). Cyclin A localizes to the nucleus from S-phase to nuclear envelope breakdown (NEBD), whereas cyclin B1 accumulates in the cytoplasm in G2 and only enters the nucleus shortly before NEBD (Minshull et al., 1990; Pines and Hunter, 1991). Once the nuclear envelope has broken down, cyclin A is rapidly destroyed, while cyclin B1 associates with the condensed chromosomes and the spindle apparatus and is stabilized by the spindle checkpoint (Pines and Hunter, 1991). These differences are thought to determine substrate specificity in vivo, despite CDK1-cyclin A and B complexes having very similar substrate phosphorylation characteristics in vitro (Minshull et al., 1990). CDK1 activity was later shown to be required for spindle checkpoint signaling (D’Angiolella et al., 2003), although the requirement for a specific cyclin was not determined. Subsequently, it was found that cyclin B1 also localizes to kinetochores, suggesting that CDK1-cyclin B1 may play specific roles in checkpoint function or the regulation of microtubule attachments (Bentley et al., 2007; Chen et al., 2008). The related cyclin B2 is present at the endoplasmic reticulum and Golgi apparatus and mediates mitotic regulation of these organelles (Jackman et al., 1995; Draviam et al., 2001). How cyclin B1 and B2 localize to these different structures remains unclear. Cyclin B1 has been reported to interact with separase (Gorr et al., 2005) and spindle checkpoint proteins (Pagliuca et al., 2011); however, none of these proteins have been shown to contribute to cyclin B1 localization. In the case of separase, the cyclin B1 interaction is direct and important for maintaining separase inhibition until the onset of anaphase (Gorr et al., 2005). By contrast, the functional consequences of interactions with spindle checkpoint proteins were not mapped to a specific protein and have not been explored further.

## Results and discussion

### CCNB1 localizes to unattached kinetochores

**Figure 1.**
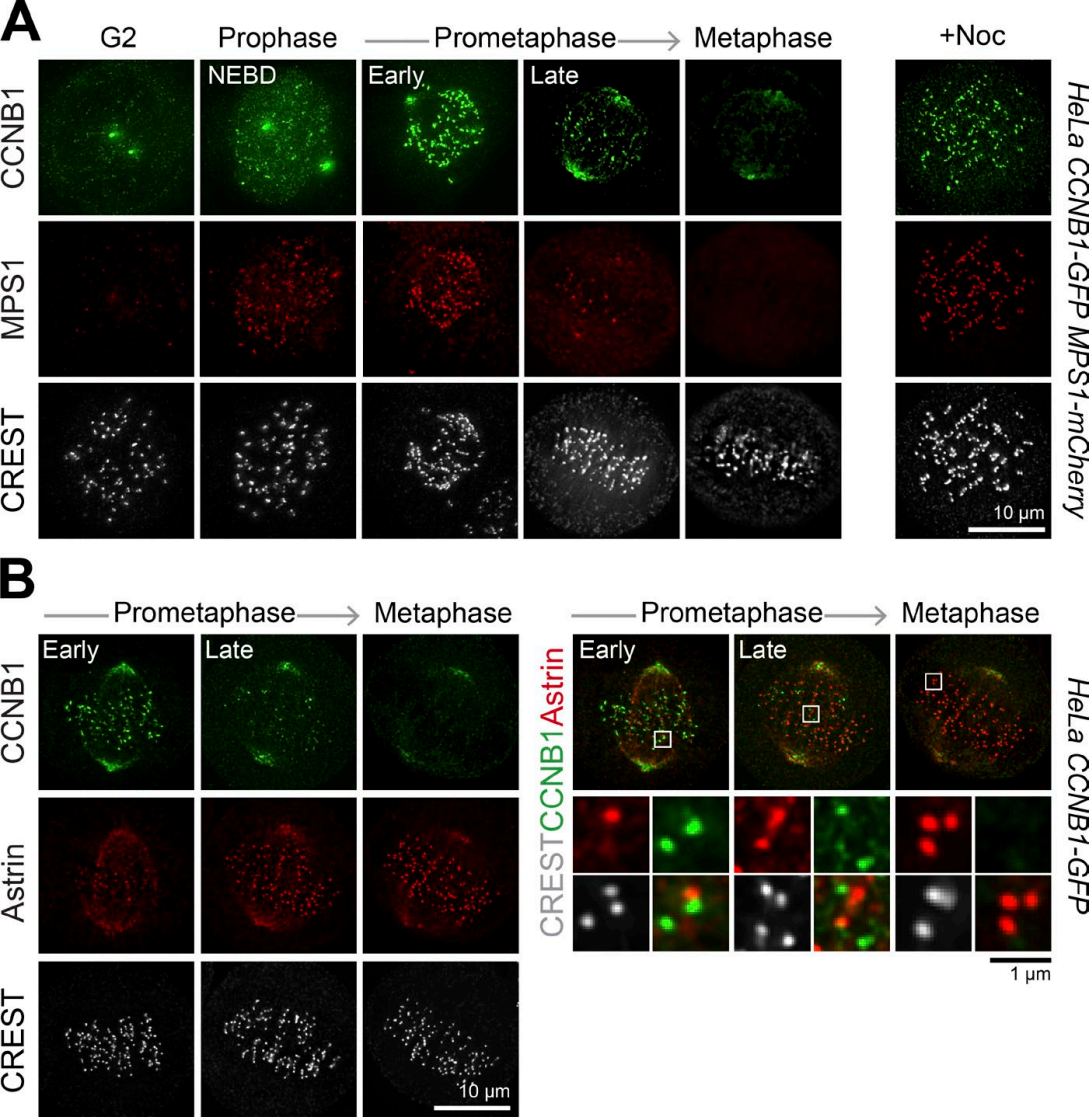
**MPS1 and CCNB1 localize to unattached kinetochores. (A)** HeLa CCNB1-GFP/MPS1-mCherry cells were stained with CREST serum to label kinetochores. Representative images of cells at different phases of mitosis and after 5-min treatment with 3 µM nocodazole (right) are shown. **(B)** HeLa CCNB1-GFP cells at the different stages of mitosis were stained with antibodies for Astrin and kinetochores (CREST).

To enable investigation of the spatial and temporal relationship between endogenous cyclin B1 (CCNB1) and MPS1, and checkpoint activation during mitotic entry, GFP and mCherry sequences were edited into the CCNB1 and MPS1 loci in HeLa and telomerase immortalized human diploid retinal pigmented epithelial cells (hTERT-RPE1) using CRISPR/Cas9 (Stewart-Ornstein and Lahav, 2016; Fig. S1 A). Imaging of these cell lines revealed that CCNB1 associated with the centrosomes in G2 cells, translocated into the nucleus, and remained associated with the spindle poles until metaphase (Fig. 1 A and Video 1). In addition, CCNB1 showed transient localization to punctate structures also labeled by MPS1 in prophase and prometaphase cells (Fig. 1 A and Video 2). Consistent with the idea that these are unattached kinetochores, the signals were maximal in nocodazole-treated cells (Fig. 1 A) and were mutually exclusive with Astrin (Figs. 1 B and S1 B), a marker for attached kinetochores (Schmidt et al., 2010). The presence of CDK1 subunits at the same localization as CCNB1 (Fig. S1 C) suggested that kinetochore-localized CCNB1 is likely to represent an activated pool of CDK1-CCNB1. By contrast, CCNA2, the other abundant cyclin associated with entry into mitosis (Di Fiore and Pines, 2010; Pagliuca et al., 2011), did not localize to kinetochores and was rapidly destroyed during the period of checkpoint activation in CCNA2-GFP CRISPR-edited cells (Fig. S1 D). This differential localization suggests that CCNB1 has specific functions and interaction partners at kinetochores that are not shared with CCNA2.

### MPS1 and CCNB1 localization to kinetochores is codependent

**Figure 2.**
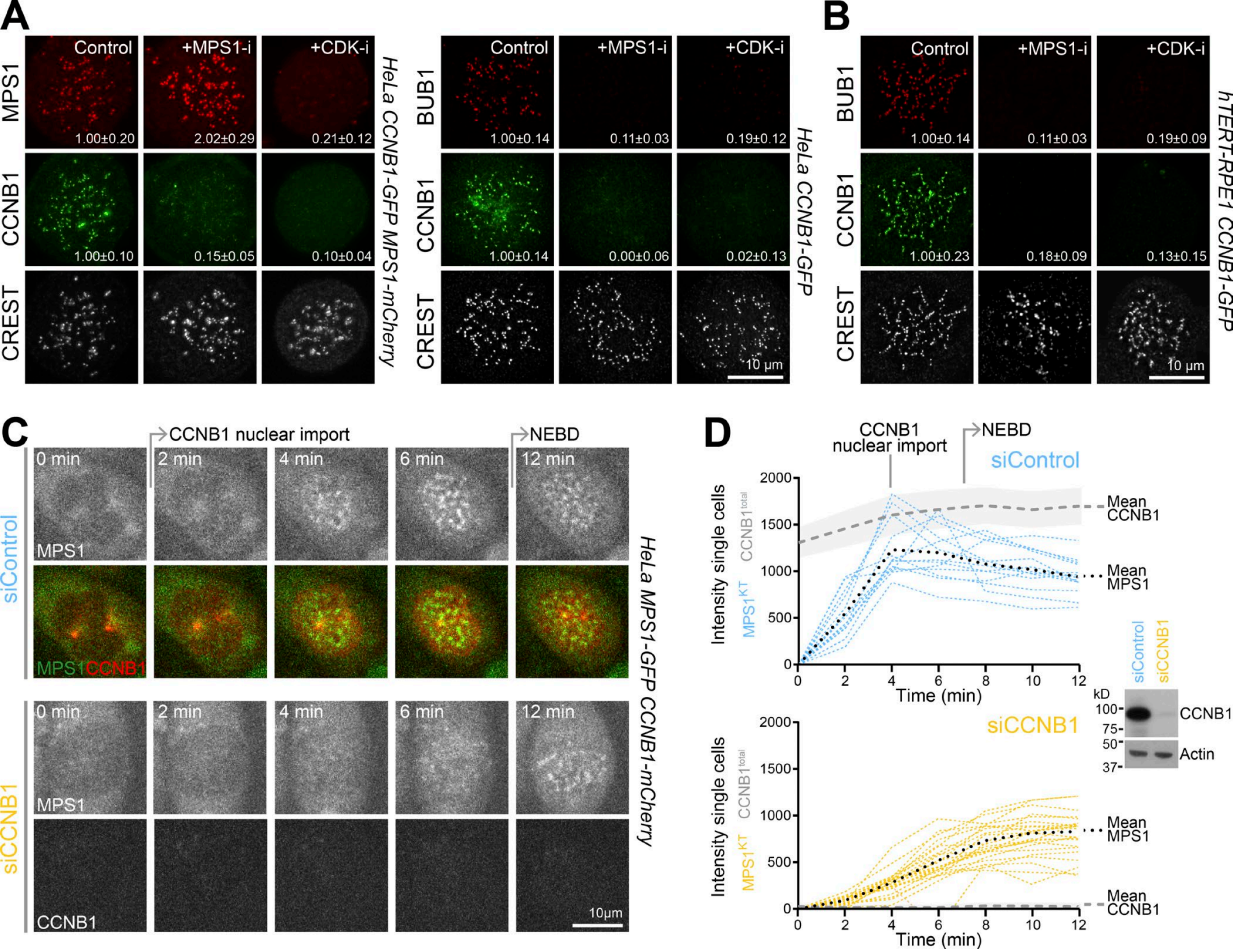
**MPS1 and CDK regulate CCNB1 kinetochore localization. (A and B)** HeLa CCNB1-GFP/MPS1-mCherry (A, left), HeLa CCNB1-GFP (A, right), or hTERT1-RPE1 CCNB1-GFP (B) cells were arrested in mitosis with 0.3 µM nocodazole for 4 h and then treated with 2 µM AZ3146 (+MPS1-i) or 5 µM flavopiridol (+CDK-i) for 10 min. Kinetochores were stained for CREST and BUB1. The numbers inset in the image panels indicate the mean kinetochore signal ± SEM of MPS1, BUB1, and CCNB1 relative to the control (15 kinetochores per cell and 12 cells in each of three independent experiments). **(C)** Control (siControl) or CCNB1 (siCCNB1) depleted HeLa MPS1-GFP/CCNB1-mCherry cells were imaged every 2 min. Representative images of cells from the point at which cell rounding was first observed, set to 0 min, are shown. **(D)** Fluorescence intensity (I_t_) for total cellular CCNB1-mCherry (CCNB1^total^) and MPS1-GFP at kinetochores (MPS1^KT^) are plotted over time for single cells (*n* = 13 for siControl and 22 for siCCNB1). Mean CCNB1-mCherry signal is indicated with a gray dashed line; the light gray area marks the SEM. For MPS1-GFP, color-coded lines show the kinetochore signal from individual cells as a function of time, and the black dots mark the mean intensity. Western blot of the siControl and siCCNB1 cells confirmed depletion of CCNB1; actin was used as a loading control.

Recruitment of the spindle checkpoint proteins to unattached kinetochores is dependent on MPS1 activity (Tighe et al., 2008; Hewitt et al., 2010; Santaguida et al., 2010). To test if this requirement also applies to CCNB1, CCNB1 and MPS1 CRISPR-edited HeLa or hTERT1-RPE1 cells were treated with MPS1 inhibitors. When MPS1 was inhibited, both CCNB1 and BUB1 were lost from kinetochores within 10 min (Fig. 2, A and B, control and +MPS1-i). Conversely, in the same time period, MPS1 levels at kinetochores increased twofold (Fig. 2, A and B, control and +MPS1-i). This effect was reported previously (Hewitt et al., 2010), and while the mechanism is not fully understood, MPS1 N-terminal autophosphorylation has been shown to promote its release from kinetochores (Wang et al., 2014). Taken together, these results are most consistent with the idea that CCNB1 is binding to an MPS1-dependent receptor at the kinetochore and not directly to MPS1. CCNB1 therefore has properties similar to bona fide spindle checkpoint proteins with respect to its localization and dependence on MPS1 activity. This MPS1-dependent kinetochore-localized pool of CDK1-CCNB1 creates a potential positive feedback loop within the checkpoint through phosphorylation of MPS1 and BUB1 (Ji et al., 2017; Qian et al., 2017; Zhang et al., 2017; Hayward et al., 2019; and see Hayward et al. in this issue). In agreement with this idea, upon CDK inhibition, MPS1, BUB1, and CCNB1 were lost from kinetochores within 10 min (Fig. 2, A and B, control and +CDK-i). Feedback between CDK1-CCNB1 and MPS1 activities therefore creates a mutual dependence for localization to unattached kinetochores. Such a feedback loop would in principle facilitate rapid establishment of a checkpoint signal during mitotic entry and modulate the properties of the checkpoint during mitotic exit, and these ideas were explored further.

Cells depleted of CCNB1 enter mitosis but have a strongly impaired spindle checkpoint and fail to arrest in the presence of nocodazole (Chen et al., 2008; Gong and Ferrell, 2010). To test if this is due to a failure to rapidly establish a checkpoint signal, the recruitment of MPS1 to kinetochores was examined as cells entered mitosis under control or CCNB1-depleted conditions. In agreement with this idea, a burst of MPS1 recruitment to kinetochores was concomitant with nuclear import of CCNB1 and reached a maximum after 4 min in control cells (Fig. 2, C and D, siControl). The kinetochore pool of MPS1 then dropped slightly at NEBD, possibly owing to the reduction in CCNB1 and MPS1 concentrations when cytoplasm and nuclear volumes mix. In the absence of CCNB1, MPS1 recruitment to kinetochores was attenuated and rose slowly to a maximum after 10–12 min (Fig. 2, C and D, siCCNB1). Together, these results show that CDK1-CCNB1 is necessary for the rapid establishment of a checkpoint signal at unattached kinetochores as cells enter mitosis.

### PP2A-B55 sets the CCNB1 threshold for checkpoint signaling

**Figure 3.**
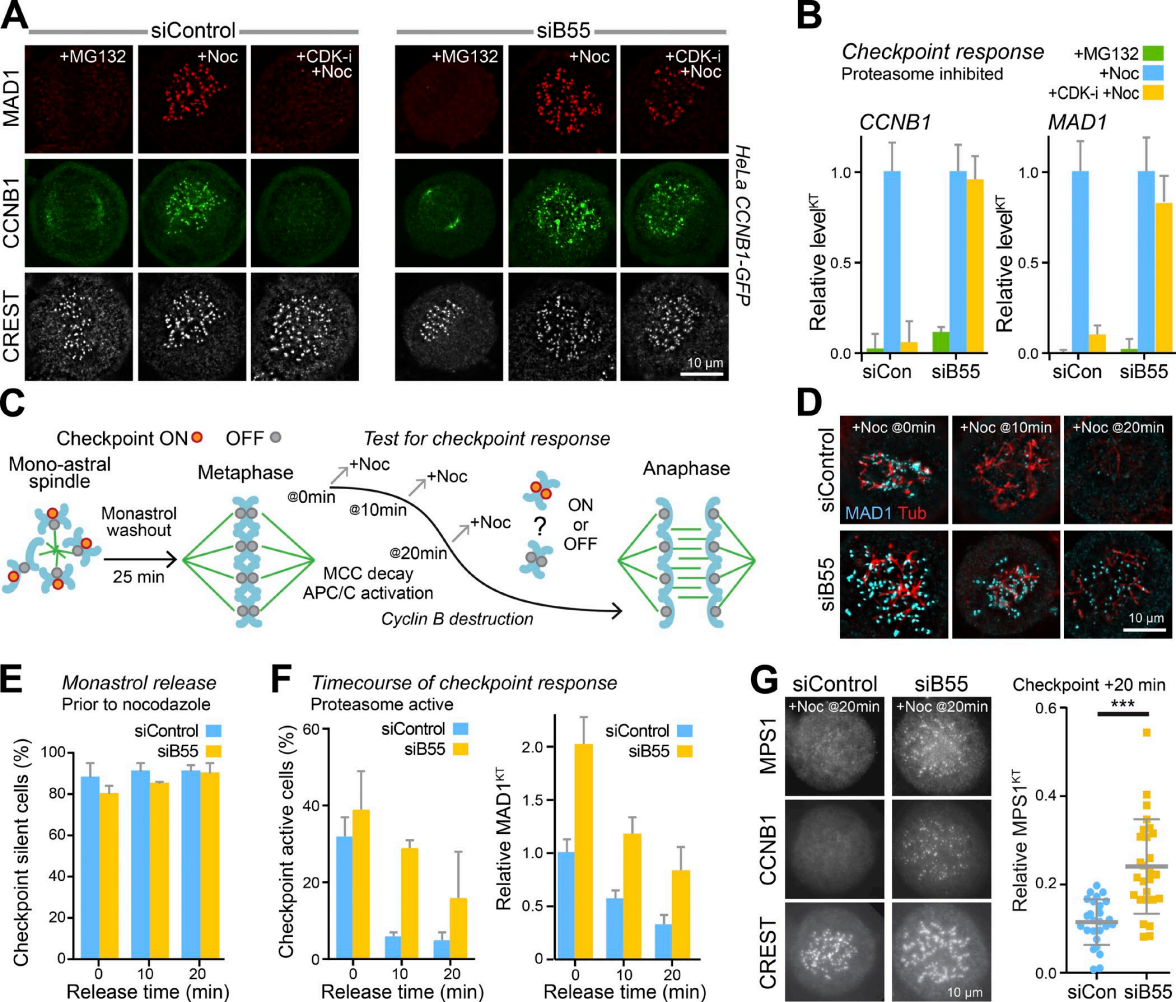
**PP2A-B55 opposes CCNB1 recruitment to kinetochores. (A)** Checkpoint signaling and CCNB1 localization were followed in control (siControl) and PP2A-B55 (siB55) depleted HeLa CCNB1-GFP cells. Cells were arrested for 2.5 h with 20 µM MG132, and then either fixed immediately (+MG132) or treated with 3 µM nocodazole for 5 min (+Noc) or with 5 µM flavopiridol for 1 min followed by addition of 3 µM nocodazole for 5 min (+CDK-i +Noc). MAD1 and kinetochores (CREST) were detected using antibodies, and CCNB1 using GFP fluorescence. **(B)** Mean kinetochore intensity ± SEM of CCNB1 and MAD1 in control (siCon) and B55-depleted (siB55) cells are plotted (15 kinetochores per cell for ≥5 cells in each of three independent experiments). **(C)** A schematic of the checkpoint response assay. **(D)** Control (siControl) and PP2A-B55 (siB55) depleted HeLa cells arrested in mitosis for 3 h with 100 µM monastrol were washed into fresh growth medium for 25 min to allow spindle formation. At that point, 0 min, or after a further 10 or 20 min, cells were challenged with 3 µM nocodazole for 5 min to test for the checkpoint response, fixed, and then stained for MAD1 and tubulin. **(E)** Graphs show the fraction of checkpoint silenced cells at different times after monastrol washout, before nocodazole addition. Bars indicate the SEM (for siControl: 0/10/20 min *n* = 263/293/277 and for siB55: *n* = 296/294/277). **(F)** Graphs show the fraction of checkpoint active cells with unseparated sister chromatids (for siControl: 0/10/20min *n* = 232/302/297 and for siB55: *n* = 277/257/306) and MAD1 signal at kinetochores (for siControl: 0/10/20min *n* = 64/48/65 and for siB55: *n* = 46/62/49); error bars indicate the SEM. **(G)** CCNB1 and MPS1 localization are shown for the 20-min time point challenged with 3 µm nocodazole (+Noc) in control (siCon) or PP2A-B55 (siB55) depleted HeLa CCNB1-GFP/MPS1-mCherry cells. MPS1 intensity is plotted relative to the cytoplasmic CCNB1 signal for individual kinetochores with the mean and SD (15 kinetochores per cell in 26 [siCon] or 27 [siB55] cells).

During mitotic exit, the point of no return for MPS1-dependent checkpoint signaling is determined by the balance of activities of CDK1 and a CDK-inhibited counteracting phosphatase PP2A-B55 (Hayward et al., 2019). This regulation extends to CCNB1, as cells depleted of PP2A-B55 showed recruitment of CCNB1 to kinetochores following nocodazole addition in the absence of ongoing CDK-activity (Fig. 3, A and B, siB55). In contrast, in control cells, the kinetochore pools of CCNB1 and MAD1 were dependent on ongoing CDK activity (Fig. 3, A and B, siControl). This is due to PP2A-B55 reactivation following CDK1 inhibition. CDK1 and PP2A-B55 thus act antagonistically to control the localization of CCNB1 to unattached kinetochores. In this regard, CCNB1 has properties similar to that of MPS1 and bona fide spindle checkpoint proteins such as MAD1. The relationship between CCNB1 and PP2A-B55 and its role in checkpoint regulation during mitotic exit was then investigated further. To do this, synchronized checkpoint silenced metaphase cells produced by 25-min washout from monastrol were challenged with 3 µM nocodazole at different times during mitotic exit (Fig. 3 C). In control cells, before nocodazole addition, the checkpoint was silenced to an equivalent extent at all time points and a checkpoint response defined by MAD1-positive kinetochores was only seen at the earliest point of challenge with nocodazole (Fig. 3 D–F, siControl). From an equivalent checkpoint silenced state, the potential to generate MAD1-positive kinetochores and elicit a checkpoint response was retained for >20 min in PP2A-B55–depleted cells (Fig. 3, D–F, siB55). PP2A-B55 thus plays a role in setting the length of the checkpoint-responsive window at the metaphase-anaphase transition. CCNB1 and MPS1 localizations were then examined at 20 min, the last time point, when CCNB1 levels are approaching their minimum. This revealed that both CCNB1 and MPS1 were recruited to kinetochores in PP2A-B55 depleted cells but not control cells (Fig. 3 G). In the absence of PP2A-B55, CCNB1 and MPS1 localize to unattached kinetochores and therefore elicit a checkpoint signal at lower CCNB1 concentrations. These findings suggest that there is an MPS1-dependent kinetochore receptor for CCNB1 which together with MPS1 and CDK1-CCNB1 forms part of a positive feedback loop at unattached kinetochores opposed by the action of PP2A-B55.

### MAD1 is the MPS1-dependent kinetochore receptor for CCNB1

**Figure 4.**
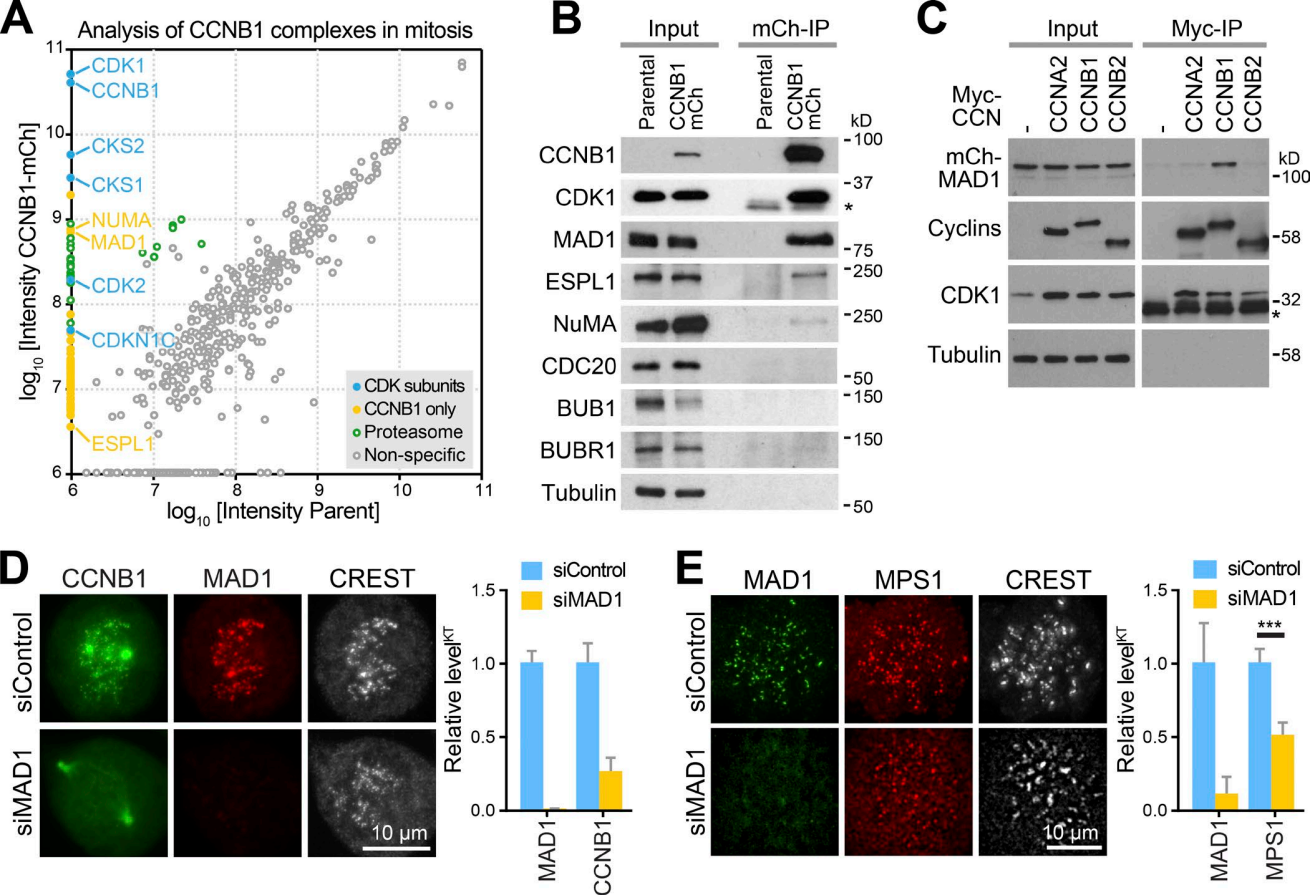
**MAD1 is the kinetochore receptor for CCNB1. (A and B)** CCNB1-mCherry (CCNB1-mCh) was immunoprecipitated (IP) with anti-mCherry antibodies from CCNB1-mCh or parental HeLa cells arrested in mitosis. Coprecipitating proteins were analyzed by mass spectrometry in three independent experiments (A) or by Western blotting (B). Intensities of proteins identified by mass spectrometry in all experiments were plotted against each other. Nonspecific components of the IPs are found equally in both samples and therefore align along the diagonal. Specific components of CCNB1 complexes cluster along the y-axis of the plot. **(C)** Cyclin IPs were performed from HEK293T cells cotransfected with full-length mCh-MAD1 and Myc-CCNA2, CCNB1 or CCNB2 constructs indicated. Myc IPs were Western blotted for Myc to detect precipitated cyclins, for CDK1, and for mCh to detect coprecipitated MAD1. Asterisks mark the antibody light chain detected in the CDK1 blots for IP samples. **(D)** Prometaphase localization of CCNB1 in control (siControl) and MAD1 (siMAD1) depleted HeLa CCNB1-GFP cells arrested with 20 µM MG132 for 30 min, then stained for MAD1 and kinetochores (CREST). Kinetochore fluorescence of MAD1 and CCNB1 normalized to the respective mean signal in control cells and SEM are plotted in the bar graphs (15 kinetochores per cell and 12 cells in each of three independent experiments). **(E)** Prometaphase localization of MPS1 in control (siControl) and MAD1 (siMAD1) depleted HeLa MPS1-mCherry cells stained for MAD1 and kinetochores (CREST). Kinetochore fluorescence of MAD1 and MPS1 normalized to the respective mean signal in control cells and SEM are plotted in the bar graphs (*n* = 19, P value for MPS1-mCherry < 0.0001, Student’s *t* test).

To identify the MPS1-dependent kinetochore receptor for CCNB1, CCNB1 complexes were isolated from HeLa CCNB1-mCherry or CCNB1-GFP CRISPR-edited cells and the parental cell line under conditions where CCNB1 was localized to unattached kinetochores. Comparison of these samples by mass spectrometry showed that CCNB1 interacts with the expected mitotic kinase components, CDK1 and CKS1/2 (Fig. 4 A and Table S1) and substoichiometric amounts of the CDK-inhibitor p57^KIP2^ (CDKN1C; Lee et al., 1995). Most important for this work, the kinetochore localized checkpoint protein MAD1 was highly enriched in CCNB1 complexes isolated from either the CCNB1-mCherry or -GFP cell lines (Fig. 4, A and B; and Fig. S2 A). Because the interaction was not dependent on ongoing MPS1 activity (Fig. S2 B), we conclude that MPS1 regulates the recruitment of preexisting cytosolic MAD1-CCNB1 complexes to unattached kinetochores, rather than directly promoting the interaction of MAD1 with CCNB1. This idea is consistent with the MPS1-dependent recruitment of MAD1 to kinetochores by BUB1 (Ji et al., 2017; Qian et al., 2017; Zhang et al., 2017). The known CCNB1 interaction partner separase (ESPL1; Gorr et al., 2005) and the spindle protein NuMA (Lydersen and Pettijohn, 1980) were also identified by this approach (Fig. 4, A and B; and Fig. S2 A); however, neither of these localize to kinetochores. Since MAD1 was not enriched above the negative control in either CCNA2 or CCNB2 immunoprecipitations (Fig. 4 C), we conclude that MAD1 interaction is a specific property of CCNB1. Direct support for the idea that MAD1 is the MPS1-regulated kinetochore receptor for CCNB1 came from the observation that CCNB1 failed to localize to kinetochores in cells depleted of MAD1 yet remained on spindle poles (Fig. 4 D). Furthermore, depletion of MAD1 also reduced the level of MPS1 at kinetochores by 50% (Fig. 4 E), consistent with the notion that MAD1 is part of a positive feedback loop between CDK1-CCNB1 and MPS1.

**Figure 5.**
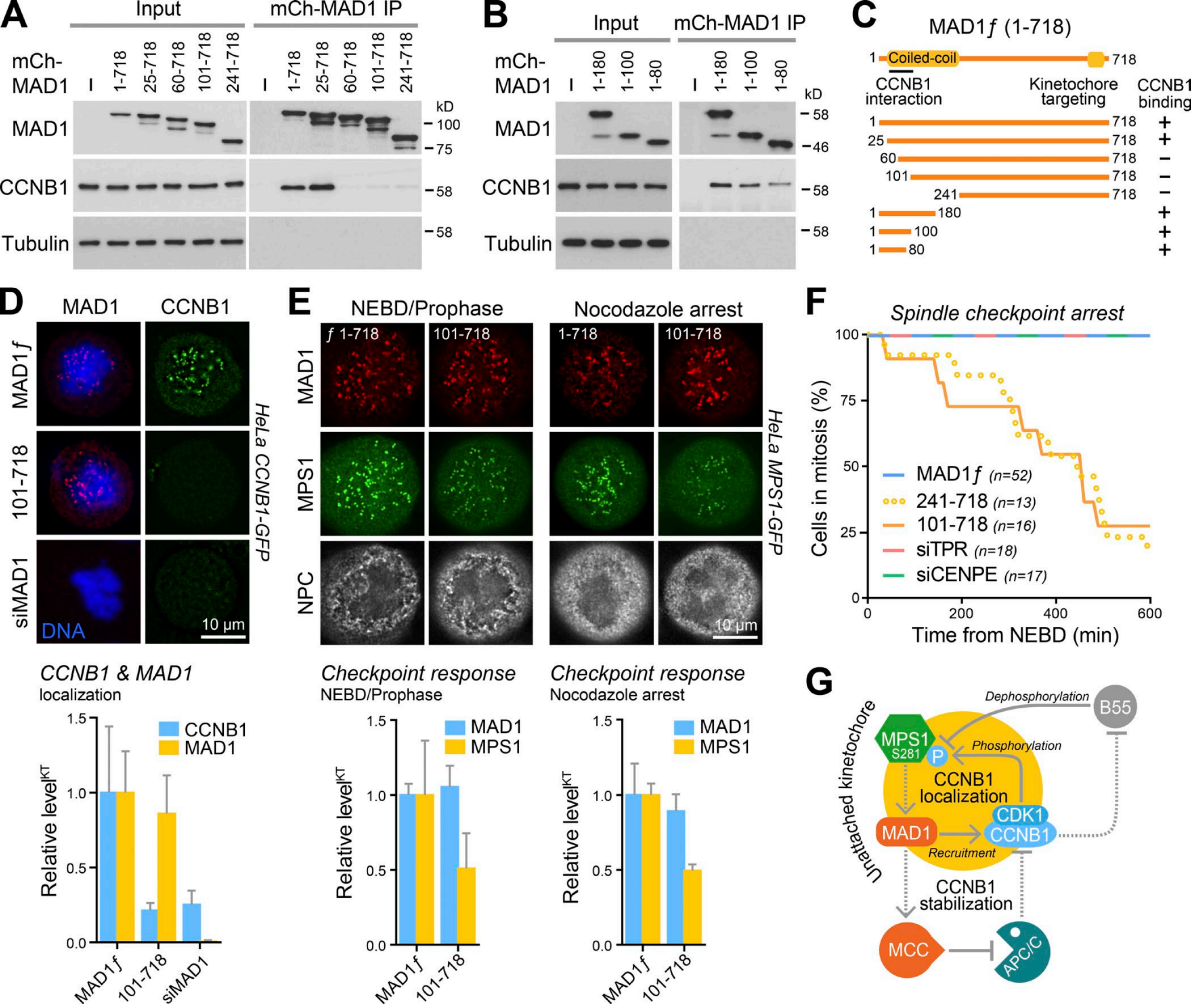
**Efficient checkpoint arrest requires the interaction of CCNB1 and MAD1. (A and B)** HeLa cells were transfected with Myc-CCNB1 and either empty vector negative control (–), full-length mCh-MAD1, N-terminal (A) or C-terminal (B) MAD1 truncations. MAD1 IPs were performed using mCh-antibodies and Western blotted for CCNB1 with Myc-antibodies. **(C)** A schematic of the MAD1 truncations summarizes the CCNB1 binding data. **(D)** mCh-MAD1^ƒ^ or MAD1^101–718^ were expressed in HeLa CCNB1-GFP cells depleted of endogenous MAD1 for 72 h with an siRNA duplex targeting the 3′ UTR of the MAD1 mRNA (siMAD1). Cells were then arrested in mitosis for 4 h with 0.3 µM nocodazole and 20 µM MG132. Mean kinetochore fluorescence ± SEM of MAD1 and CCNB1 normalized to the respective signal in control cells are plotted in the graph (*n* ≥ 12 cells per condition, 15 kinetochores per cell in three independent experiments). DNA was stained with DAPI. **(E)** mCh-MAD1^ƒ^ or MAD1^101–718^ were expressed in HeLa MPS1-GFP cells depleted of endogenous MAD1 for 72 h with an siRNA duplex targeting the 3′ UTR of the MAD1 mRNA. Cells were then arrested in mitosis for 4 h with 0.3 µM nocodazole and 20 µM MG132 and stained for nuclear pore complexes (NPC). Graphs show the mean MAD1 or MPS1 levels ± SEM at unattached kinetochores (15 kinetochores per cell) in normal prophase (*n* = 3 cells per condition) and nocodazole-arrested mitosis (*n* = 12 cells per condition). **(F)** HeLa Flp-in/TREx GFP-MAD1^ƒ^, GFP-MAD1^241–718^, and GFP-MAD1^101–718^ cells were depleted of endogenous MAD1 for 72 h. Expression of GFP-MAD1 transgenes was induced for the last 48 h, and the cells were then imaged over 10 h in the presence of 0.3 µM nocodazole. The proportion of cells remaining in mitosis over a 600-min time course is plotted. HeLa cells depleted of CENP-E or TPR were checkpoint challenged and imaged in the same way. **(G)** MPS1 promotes recruitment of MAD1 to unattached kinetochores (shown as the yellow circle). CDK1-CCNB1 localizes to unattached kinetochores through an interaction with MAD1, creating a positive feedback loop counteracted by the action of PP2A-B55 on MPS1. MAD1 promotes formation of the APC/C inhibitor, mitotic checkpoint complex (MCC). This creates a second positive feedback loop preventing CCNB1 destruction. Solid lines indicate direct interactions (recruitment, phosphorylation, or dephosphorylation), whereas dotted lines indicate multistep processes. Components drawn outside the yellow circle are active globally rather than acting locally at the unattached kinetochore.

To test this latter proposal, it was necessary to create a CCNB1 binding–defective form of MAD1 and test its ability to support MPS1 recruitment and spindle checkpoint arrest. N-terminal truncation of MAD1 resulted in a loss of CCNB1 interaction, and a putative binding site was mapped to amino acids 25–100 (Fig. 5 A). N-terminal fragments of MAD1 encompassing this region were sufficient for the CCNB1 interaction (Fig. 5 B). Thus, the first 100 amino acids of MAD1 are both necessary and sufficient for CCNB1 binding (Fig. 5 C), separate from the C terminus important for kinetochore localization (Kim et al., 2012). Cells depleted of MAD1 lose CCNB1 from kinetochores (Fig. 5 D, siMAD1), and this can be rescued by expression of full-length MAD1 (Fig. 5 D, MAD1ƒ). In contrast, MAD1 lacking the first 100 amino acids localized to kinetochores but failed to recruit CCNB1 (Fig. 5 D, MAD1^101–718^). Furthermore, compared with full-length MAD1, cells rescued with MAD1^101–718^ recruited significantly less MPS1 to kinetochores both early in mitosis in prophase shortly after NEBD and later in mitosis in the presence of nocodazole when the checkpoint signal should be maximal (Fig. 5 E). These observations support the idea that there is a positive feedback loop created by MAD1-dependent recruitment of CCNB1 that promotes MPS1 localization. The functional consequences of the loss of this interaction for the spindle checkpoint were then investigated. Synchronized cells depleted of MAD1 entered mitosis with similar timing to matched controls (Fig. S3 A), but then following NEBD failed to stabilize CCNB1 and rapidly progressed into anaphase (Fig. S3, B and C). If MAD1 depletion was rescued using wild-type MAD1, the cells showed a robust checkpoint arrest when challenged with nocodazole (Fig. 5 F, MAD1ƒ). In contrast, rescue with MAD1^241–718^ or MAD1^101–718^, which fail to interact with and promote CCNB1 recruitment to kinetochores, did not support an efficient checkpoint arrest (Fig. 5 F). TPR and CENP-E have previously been reported as interactors with the N terminus of MAD1. However, no differences in TPR or CENP-E staining during mitosis were observed when endogenous MAD1 was replaced by MAD1^101–718^ (Fig. S3, D and E). Moreover, depletion of either of these proteins did not affect spindle checkpoint arrest under the conditions of our assay (Figs. 5 F and S3 F), indicating that the loss of these interaction partners is unlikely to be the cause of the observed SAC defect. In summary, these findings support the view that CCNB1 is an important interaction partner of the MAD1 N terminus required for robust spindle checkpoint arrest.

### A central role for CDK1-CCNB1 in the spindle checkpoint

The molecular basis for the requirement for CDK activity in spindle checkpoint signaling (D’Angiolella et al., 2003) has remained elusive due to the multiple roles played by CDK–cyclin complexes in mitosis. Here, we show that CCNB1 is an intrinsic part of the spindle checkpoint and localizes to unattached kinetochores, where it directly contributes to a MAD1-dependent positive feedback loop sustaining MPS1 activation (Fig. 5 G). In addition, in an accompanying study, we report that MPS1 recruitment to unattached kinetochores and generation of a checkpoint response is enabled by CDK1-CCNB1 and counteracted by the phosphatase PP2A-B55 (Hayward et al., 2019). These two mechanisms, CDK1-CCNB1–dependent inhibition of PP2A-B55 and concentration of CDK1-CCNB1 at unattached kinetochores, reinforce one another to promote timely and efficient MPS1 localization, and thus checkpoint signaling. Furthermore, we propose that the pool of CDK1-CCNB1 at unattached kinetochores is also necessary for efficient phosphorylation of other CDK targets critical for spindle checkpoint signaling. Key among these substrates is BUB1 S459 phosphorylation by CDK1, which mediates MAD1 recruitment (Ji et al., 2017; Qian et al., 2017; Zhang et al., 2017). Although further work is needed to define the full extent of CDK1-CCNB1 substrates at kinetochores and centromeres and to explore the properties of the MPS1-MAD1-CCNB1 positive feedback loop, these findings help to explain the requirement for CCNB1 in robust checkpoint arrest by placing it at the heart of the spindle checkpoint surveillance system.

## Materials and methods

### Reagents and antibodies

General laboratory chemicals and reagents were obtained from Sigma-Aldrich and Thermo Fisher Scientific unless specifically indicated. Inhibitor stocks prepared in DMSO were as follows: 5 mM CDK-inhibitor flavopiridol (Sigma-Aldrich), 20 mM MPS1-inhibitor AZ3146 (Tocris Bioscience), 100 mM Eg5 inhibitor Monastrol (Cambridge Bioscience), 20 mM proteasome inhibitor MG132 (Insight Bioscience), and 6 mM microtubule polymerization inhibitor nocodazole (Merck). Thymidine (Sigma-Aldrich; 100 mM stock) and doxycycline (InvivoGen; 2 mM stock) were dissolved in water. A 1 mM stock of the DNA vital dye SiR-Hoechst (Spirochrome) was prepared in DMSO.

Commercially available polyclonal antibodies (pAbs) or mAbs were used for BUB1 (mouse mAb; Abcam, ab54893), MAD1 (rabbit pAb; GeneTex, GTX105079), CCNB1 (mouse mAb GNS3; Millipore, 05-373), BUBR1 (rabbit pAb; Bethyl, A33-386A), CDC20 (rabbit pAb; Cell Signaling, 4823S), actin (HRP-conjugated mouse mAb AC-15; Abcam, ab49900), tubulin (mouse mAb DM1A; Sigma-Aldrich, T6199), NPC (mouse mAb414; Abcam), NuMA (rabbit mAb; Abcam, ab109262), HEC1 (mouse mAb 9G3.23; GeneTex, GTX70268), c-Myc epitope tag (mouse mAb 9E10; Sigma-Aldrich), FLAG epitope tag (mouse mAb FG4R; Thermo Fisher Scientific, MA1-91878; and rabbit pAb; Sigma-Aldrich, F7425), GFP (rabbit pAb; Abcam, ab290), and mCherry (rabbit pAb; Abcam, ab167453). Human CREST serum was obtained from Antibodies Inc. (15-234-0001). Antibodies to Separase (ESPL1) and MPS1 were raised in sheep against recombinant His-tagged fragments of Separase (amino acids 1876–2120) and MPS1 (amino acids 1–260) and affinity purified against the same recombinant proteins. Rabbit antibodies against Astrin and sheep antibodies against mCherry and GFP have been described previously (Thein et al., 2007; Bastos and Barr, 2010). Secondary donkey antibodies against mouse, rabbit, or sheep and labeled with Alexa Fluor 488, Alexa Fluor 555, Alexa Fluor 647, Cy5, or HRP were purchased from Molecular Probes and Jackson ImmunoResearch Laboratories, respectively. Affinity-purified primary and HRP-coupled secondary antibodies were used at 1 µg/ml final concentration. For Western blotting, proteins were separated by SDS-PAGE and transferred to nitrocellulose using a Trans-blot Turbo system (Bio-Rad). Protein concentrations were measured by Bradford assay using Protein Assay Dye Reagent Concentrate (Bio-Rad). All Western blots were revealed using ECL (GE Healthcare).

### Molecular biology

All DNA primers were obtained from Invitrogen. Human CDK1, CCNB1, CCNB2, CCNA2, and MAD1 were amplified using Pfu polymerase (Promega). Mammalian expression constructs were made in pcDNA5/FRT/TO and pcDNA4/TO vectors (Invitrogen) modified to encode eGFP mCherry, C-Myc (three copies), or FLAG (single copy) tags. Mutagenesis was performed using the QuickChange method (Agilent Technologies). siRNA duplexes or optimized siRNA pools were obtained from GE Healthcare. CCNB1 was depleted using pool L-003206-00. PP2A regulatory subunit B55 was depleted using a combination of four pools against each isoform. L-004824-00, L-003022-005, L-019167-00, and L-032298-00 for PPP2R2A, PPP2R2B, PPP2R2C, and PPP2R2D, respectively. MAD1 was depleted using the pool L-006825-00 or the single duplexes 5′-CCACAGGGCAGCAGCAUGAUU-3′ and 5′-CUGCUUGGCCUGACCUGCAUU-3′ targeting the 3′ UTR in siRNA rescue assays. CENP-E and TPR were depleted using siRNA duplexes 5′-CACGAUACUGUUAACAUGAAU-3′ (Dunsch et al., 2011) and 5′-GGAGGUUUCUAGAGAACAATT-3′, respectively.

### Cell culture procedures

HeLa cells and HEK293T were cultured in DMEM with 1% (vol/vol) GlutaMAX (Life Technologies) containing 10% (vol/vol) bovine calf serum at 37°C and 5% CO_2_. RPE1 cells were cultured in DME/F-12 (Sigma-Aldrich) supplemented with 1% (vol/vol) GlutaMAX (Invitrogen) and containing 10% (vol/vol) bovine calf serum at 37°C and 5% CO_2_. For plasmid transfection and siRNA transfection, Mirus LT1 (Mirus Bio) and Oligofectamine (Invitrogen), respectively, were used. HeLa cell lines with single integrated copies of the desired transgene were created using the T-Rex doxycycline-inducible Flp-In system (Invitrogen).

CRISPR/Cas9-edited HeLa and hTERT-RPE cell lines with inserted GFP or mCherry tags in the C termini of the CCNB1, CCNA2, and TTK/MPS1 gene products were constructed following a published protocol with some modifications (Stewart-Ornstein and Lahav, 2016). In brief, homology recombination cassettes containing the desired knock-in DNA with flanking regions of homology of 600–750 bp to the target locus were cotransfected with a version of pSpCAS9(BB; Addgene) containing the relevant guide RNAs and modified to remove puromycin resistance. The knock-in sequences harbor the EGFP or mCherry fluorescent proteins preceded by a glycine-serine rich flexible linker (GSSS repeated four times), a P2A ribosome skipping sequence, and a resistance marker (puromycin, blasticidin, or neomycin). Antibiotic-resistant clones were selected, and successful modification was confirmed by blotting.

### Immunofluorescence microscopy

Cells were fixed with either PTEMF (20 mM Pipes-KOH, pH 6.8, 0.2% [vol/vol] Triton X-100, 1 mM MgCl_2_, 10 mM EGTA, and 4% [wt/vol] formaldehyde) or 3% (wt/vol) paraformaldehyde in PBS followed by quenching with 50 mM NH_4_Cl in PBS and a 5-min cell permeabilization with 0.2% (vol/vol) Triton X-100 in PBS. Antibody dilutions were performed in PBS with 3% (wt/vol) BSA, except for anti-CDK1 antibodies that were diluted in PBS, 3% (wt/vol) BSA, and 0.2% (vol/vol) Triton X-100. Samples seeded on #1 thickness coverslips were imaged on a DeltaVision Core light microscopy system (GE Healthcare) using either a 60×/1.35 NA or 100×/1.4 NA objective fitted to an Olympus IX-71 microscope. Standard filter sets for DAPI (excitation 390/18 nm, emission 435/48 nm), FITC (excitation 475/28 nm, emission 525/48 nm), TRITC (excitation 542/27 nm, emission 597/45 nm), and Cy-5 (excitation 632/22 nm, emission 676/34 nm) were used to sequentially excite and collect fluorescence images on a CoolSnap HQ2 CCD camera (Photometrics) using the software package softWoRx (GE Healthcare). Cells were imaged using a 0.2-µm interval and a total stack of 2 µm and deconvolved for presentation using softWoRx. Image stacks were imported into Fiji (Schindelin et al., 2012) for maximum-intensity projection and saving as 8-bit TIFF files. TIFF files were imported into Illustrator CS6 (Adobe) for figure production. For quantification, imaging was performed using either a 60×/1.35 NA oil-immersion objective or a 40×/0.75 NA air objective on a BX61 Olympus microscope equipped with filter sets for DAPI, EGFP/Alexa Fluor 488, Alexa Fluor 555, and Alexa Fluor 647 (Chroma Technology Corp.); a CoolSNAP HQ2 camera (Roper Scientific); and MetaMorph 7.5 imaging software (GE Healthcare).

### Live-cell microscopy

Time-lapse imaging was performed using an Ultraview Vox spinning disc confocal system (Perkin Elmer) mounted on an Olympus IX81 inverted microscope with a 512 × 512–pixel EMCCD camera (ImagEM C9100-13; Hamamatsu Photonics) and Volocity software. Cells were placed in a 37°C and 5% CO_2_ environmental chamber (Tokai Hit) mounted on the microscope stage. Imaging of GFP- and mCherry-tagged proteins was performed using a 60×/1.4 NA oil-immersion objective heated to 37°C with a lens heating collar, 4–12% laser power, and 30–200-ms exposure time for 488- and 561-nm lasers, respectively. Typically, 19 planes, 0.6 µm apart, were imaged every 2 min. Maximum-intensity projection or summed projection of the fluorescent channels was performed in Fiji.

### Isolation of CCNB1 and MAD1 complexes

For CCNB1 immunoprecipitations, parental HeLa, HeLa-CCNB1-EGFP, and HeLa-CCNB1-mCherry cells were arrested with 0.3 µM nocodazole for 16 h. Pellets of 9 × 10^6^ mitotic cells were lysed in 2 ml of lysis buffer supplemented with phosphatase and protease inhibitors (20 mM Tris-HCl, pH 7.4, 150 mM NaCl, 1% [vol/vol] Igepal CA-630, 0.1% [wt/vol] sodium deoxycholate, 100 nM okadaic acid, 40 mM β-glycerophosphate, 10 mM NaF, 0.3 mM Na_3_VO_4_, protease inhibitor cocktail [Sigma-Aldrich], and phosphatase inhibitor cocktail [Sigma-Aldrich]). CCNB1-GFP or CCNB1-mCherry complexes were isolated by 2-h incubation at 4°C with 3 µg sheep-anti-GFP or anti-mCherry antibodies and 20 µl protein G–sepharose. The sepharose beads were washed twice with lysis buffer and twice with wash buffer (20 mM Tris-HCl, pH 7.4, 150mM NaCl, 0.1% [vol/vol] Igepal CA-630, 40 mM β-glycerophosphate, 10 mM NaF, and 0.3 mM Na_3_VO_4_) and either resuspended in 3× Laemmli buffer for Western blotting or eluted twice with 100 mM glycine, pH 2.6, for direct mass spectrometric analysis (Cundell et al., 2016).

To test the interaction of MAD1 with other mitotic cyclins, 10-cm dishes of HEK293T cells were transfected for 20 h with 1 µg Myc-CCNA2, CCNB1, or CCNB2 and 1 µg mCherry-MAD1 constructs. Cell lysis and immunoprecipitation was performed as before using 3 µg of 9E10 mAb to the c-Myc epitope tag for analysis by Western blotting. To map the MAD1-CCNB1 interaction, 10-cm dishes of HEK293T cells were transfected for 20 h with 1 µg Myc-CCNB1 and 1 µg mCherry-MAD1 constructs. Cell lysis and immunoprecipitation was performed as before using 3 µg of sheep anti-mCherry for analysis by Western blotting.

### MAD1 spindle checkpoint assays

T-Rex doxycycline-inducible Flp-In HeLa cells containing GFP-MAD1^1–718^ (full length), MAD1^241–718^, or MAD1^100–718^ were induced with 2 µM doxycycline for 6 h, followed by a 48-h treatment with siRNA against the 3′ UTR of MAD1. Doxycycline was added again at 2 µM 24 h into the siRNA treatment. After 48 h of treatment, the cells were synchronized by thymidine treatment and then tested for spindle checkpoint function as indicated in the figure legends.

### Measurement of protein levels at kinetochores

Image analysis was performed in Fiji using images before any deconvolution or other processing steps. Background-corrected kinetochore intensities for CCNB1, MPS1, BUB1, and MAD1 were determined by placing a 15-pixel circular region of interest (ROI) over individual kinetochores, measuring the mean pixel fluorescence, and normalizing to mean pixel intensity of the CREST channel within the same ROI. Measurements were typically performed for ≥12 cells and 15 kinetochores per cell in three or more independent experiments).

For analysis of the CCNB1 threshold for checkpoint signaling, a 40×/1.4 NA objective was used and a single stack captured. CCNB1 levels were measured in mitotic cells (counted as those with condensed chromosomes). A circular ROI was drawn around cells of interest. In the case of anaphase B cells, an ROI was drawn over one half of the dividing cell. Subsequent analysis of kinetochore intensities and CCNB1 levels was performed in Excel (Microsoft). All immunofluorescence experiments shown are representatives of at least three independent experiments, with a mean and SD derived from each independent experiment. Graphs were plotted in GraphPad Prism (GraphPad Software) using data exported from Excel.

### Online supplemental material

Fig. S1 shows Western blots of CRISPR-edited HeLa and hTERT-RPE1 cells and additional analysis of CDK-cyclin complex localization supporting Fig. 1. Additional analysis of the CCNB1 complexes described in Fig. 4 is shown in Fig. S2. Fig. S3 is an extended analysis of MAD1, CENP-E, and TPR in checkpoint signaling supplementing Fig. 5. The dynamic spindle and kinetochore localization of CCNB1 referred to in Fig. 1 is shown in Videos 1 and 2. Table S1 contains the mass spectrometry data used in Fig. 4.

## Supplementary Material

Supplemental Material (PDF)

Video 1

Video 2

Table S1
