# Supplementary material for: MAD1-dependent recruitment of CDK1-CCNB1 to kinetochores promotes spindle checkpoint signaling
Source: J Cell Biol. 2019 Jan 23;218(4):1108–17. doi: 10.1083/jcb.201808015 (PMC6446853; doi:10.1083/jcb.201808015)
Supplement: Supplemental Material (PDF) [file JCB_201808015_sm.pdf]

Supplemental material

Alfonso-Pérez et al., https://doi.org/10.1083/jcb.201808015

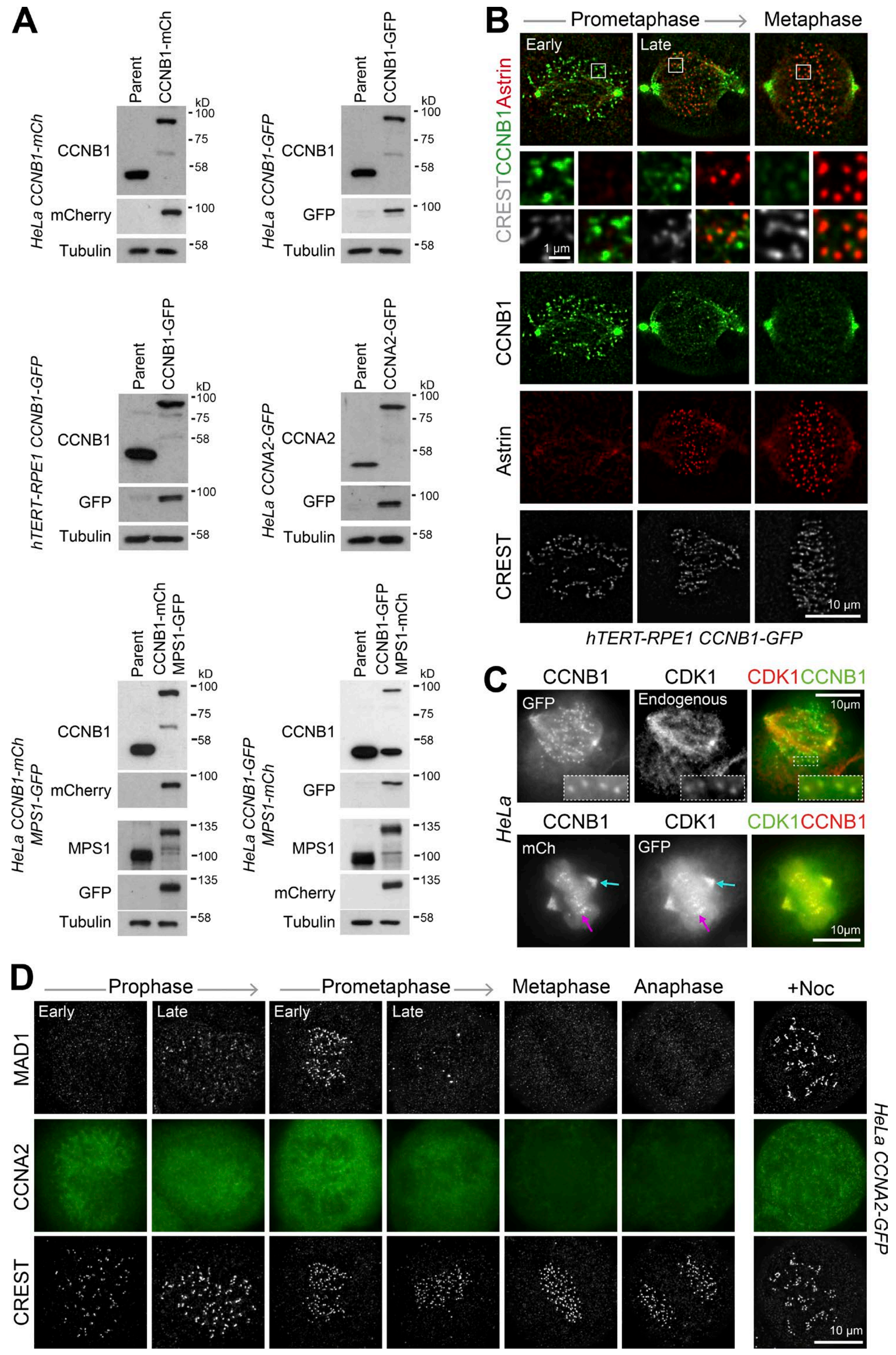

Figure S1. **CCNB1 and CDK1 localize to unattached kinetochores. (A)** Western blot analysis of HeLa and hTERT-RPE1 cells gene edited using CRISPR-Cas9 to introduce mCherry and eGFP-tags at the C terminus of CCNB1, CCNA2, and MPS1 alone or in combination as indicated in the figure. **(B)** Images of prometaphase and metaphase hTERT-RPE1 CCNB1-GFP cells stained with antibodies against Astrin and kinetochores (CREST serum) are shown. **(C)** Endogenous CDK1 was visualized with antibodies in HeLa CCNB1-GFP cells. Enlargements show a threefold magnification of the indicated areas (top). GFP-CDK1 was transiently expressed in HeLa CCNB1-mCherry cells and visualized by GFP fluorescence (bottom). CDK1 and CCNB1 at unattached kinetochores (magenta arrows) and spindle poles (cyan arrows) are indicated. **(D)** CCNA2 localization is shown in prophase, prometaphase, and metaphase HeLa CCNA2-GFP cells stained with antibodies for MAD1 and kinetochores (CREST serum). A prophase/early prometaphase cell treated with 3 µM nocodazole for 5 min (+Noc) shows a strong kinetochore signal for MAD1 but not CCNA2.

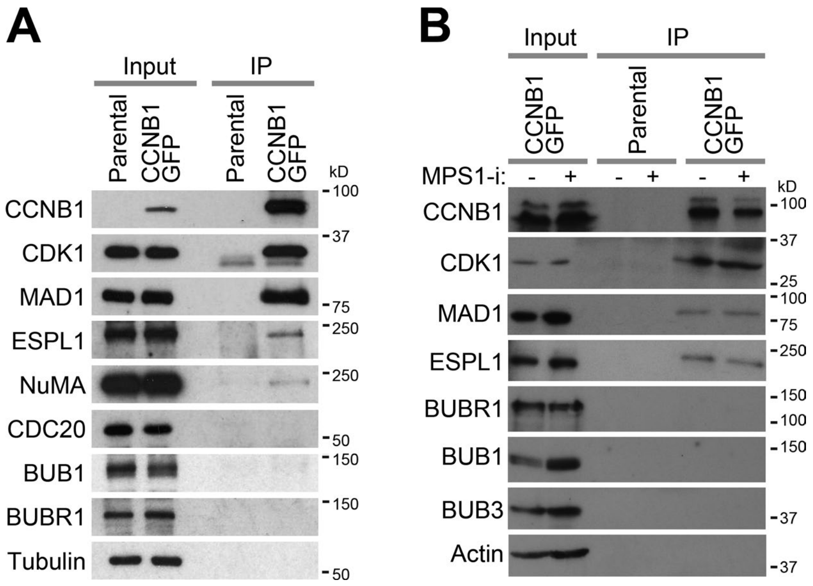

Figure S2. **CCNB1-MAD1 interaction is independent of MPS1 activity. (A)** CCNB1-GFP IPs from HeLa CCNB1-GFP cells or from parental HeLa cells arrested in mitosis for 16 h with 0.3 µM nocodazole were analyzed by blotting. **(B)** CCNB1-GFP IPs treated with 20 µM MG132 (– MPS1-i) or MG132 and 2 µM AZ3146 (+ MPS1-i) for 30 min before harvesting were analyzed by blotting.

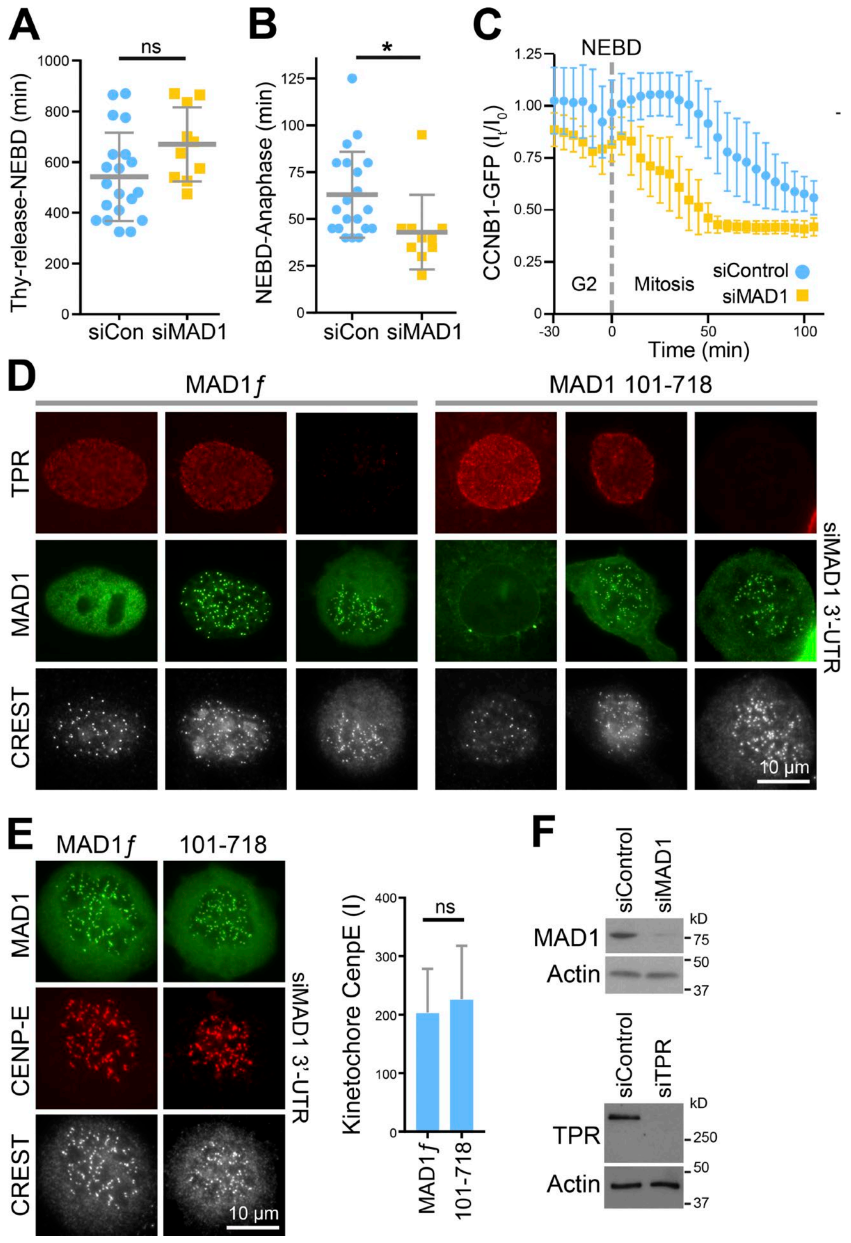

Figure S3. **Accelerated progression through mitosis in MAD1-depleted cells. (A and B)** The time taken from thymidine release to NEBD (A) and from NEBD to the onset of anaphase (B) was measured in control (siCon) and MAD1 (siMAD1) depleted HeLa CCNB1-GFP cells. Data points indicate individual cells; the mean and SD are indicated by gray bars (*n* = 20 for siControl and *n* = 10 for siMAD1). **(C)** CCNB1-GFP concentration relative to the CCNB1-GFP intensity at the onset of imaging (I_0) was measured during the same time window. Mean CCNB1-GFP signal (I_t/I_0) is plotted from −30 to +100 min relative to NEBD. **(D and E)** TPR (D) and CENP-E (E) were visualized by indirect immunofluorescence in HeLa Flp-in/TREx GFP-MAD1^{f} and GFP-MAD1$^{101–718}$ cells depleted of endogenous MAD1 and induced for the GFP-MAD1 transgenes for 24 h. The bar graph shows mean CENP-E levels at the kinetochore ± SD (*n* = 9). **(F)** Western blots of MAD1- and TPR-depleted cells were used to confirm depletion of the specific target proteins.

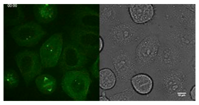

Video 1. **Related to Fig. 1.** HeLa cells expressing endogenously tagged CCNB1-GFP (green, left) are shown with brightfield images (grayscale, right). One image stack was captured every 2 min, and the video plays at 7 fps.

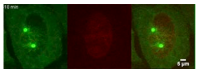

Video 2. **Related to Fig. 1.** HeLa cell expressing endogenously tagged CCNB1-GFP (green, left) with addition of 100 nM SiR-Hoechst DNA dye 8 h prior to imaging (red). One image stack was captured every 30 s, with 0 min representing the point of nuclear envelope breakdown. The video plays at 10 fps.

Provided online is one table in Excel. Table S1 contains the mass spectrometry data used in Fig. 4.
